# Asymptomatic Primary Fallopian Tube Cancer: An Unusual Cause of Axillary Lymphadenopathy

**DOI:** 10.1155/2011/402127

**Published:** 2011-12-11

**Authors:** N. A. Healy, S. O. Hynes, J. Bruzzi, S. Curran, M. O'Leary, K. J. Sweeney

**Affiliations:** ^1^Department of Surgery, School of Medicine, Clinical Science Institute, National University of Ireland, Galway, Ireland; ^2^Department of Pathology, National University of Ireland, Galway, Ireland; ^3^Department of Radiology, National University of Ireland, Galway, Ireland; ^4^Division of Obstetrics and Gynaecology, National University of Ireland, Galway, Ireland

## Abstract

Primary Fallopian tube malignancy is considered a rare disease and is often mistaken histologically and clinically for ovarian cancer. The etiology is poorly understood, and it typically presents at an advanced disease stage, as symptoms are often absent in the initial period. As a result, primary fallopian tube cancer is generally associated with a poor prognosis. We present the case of a 45-year-old female who presents with a 5-day history of left axillary swelling and a normal breast examination. Mammogram and biopsy of a lesion in the left breast revealed a fibroadenoma but no other abnormalities. Initial sampling of the axillary node was suspicious for a primary breast malignancy, but histology of the excised node refuted this. PET-CT showed an area of high uptake in the right pelvis, and a laparoscopy identified a tumor of the left fallopian tube which was subsequently excised and confirmed as a serous adenocarcinoma.

## 1. Introduction

Malignancy of the fallopian tubes is extremely rare, accounting for less than 0.2% of malignancies in women annually. Histologically and clinically, it can be difficult to differentiate from ovarian cancer, and the management of both is similar. Metastasis of fallopian tube carcinoma occurs via haematogenous, lymphatic, and peritoneal routes as well as via direct extension. However, it is rare to find extra-abdominal metastases with minimal pelvic disease. This is a case of a young woman presenting with a fallopian tube malignancy that has metastasized to an axillary lymph node. 

## 2. Case Presentation

A 45-year-old female was referred to the symptomatic breast service at the University Hospital Galway for evaluation of a left axillary swelling, which had appeared 5 days previously and had increased in size over this period. The patient also reported reduced energy levels and weight loss over the preceding 2 months. She had a history of irritable bowel syndrome but no other medical conditions. In relation to family history, there was no history of breast or ovarian cancer. On examination, a 2 cm palpable swelling was identified in the left axilla with no significant abnormalities identified in either breast.

An ultrasound (US) of the axillary mass showed a solitary, enlarged lymph node in the left axilla. A mammogram identified 2 small benign-looking lesions in the left breast and a normal right breast. Subsequent magnetic resonance imaging (MRI) identified a 14 mm left breast fibroadenoma, and the histology of a US-guided biopsy of the lesion confirmed this diagnosis.

**Figure 1:**
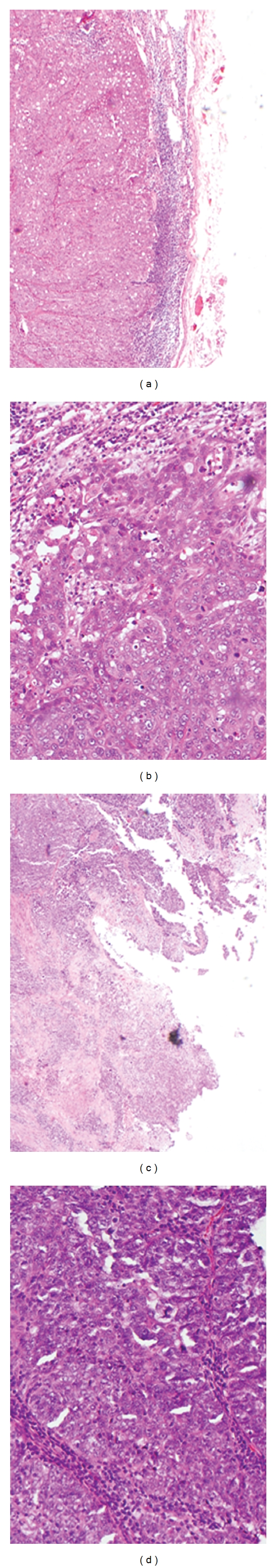
Haematoxylin and eosin-stained sections of the tumour, as the presenting metastasis seen within the lymph node ((a) and (b), 10x and 20x, resp.) and the subsequent resection of the fallopian tube ((c) and (d), 10x and 20x, resp.).

A fine needle aspiration of the left axillary lymph node was performed under ultrasound guidance. Cytology was positive for malignant cells, and these were initially suspected to be breast in origin. The axilllary node was excised under general anaesthesia, and pathology revealed a metastatic, high-grade, poorly differentiated tumor (Figure 1). In addition, the tumor was cancer antigen 125 (CA125), cancer antigen 15.3 (CA15.3), and cytokeratin 7 (CK7) strongly positive but stained negatively for estrogen receptor (ER), progesterone receptor (PR), and human epidermal growth factor receptor 2 (HER2). The tumor also showed strong nuclear positivity, with protein 53 (P53) in virtually 100% of the tumor cell nuclei. Hematological investigations revealed a markedly elevated serum CA125 of 129 U/mL (0–35 U/mL) and CA15.3 of 62. Human chorionic gonadotrophin (HCG), carcinoembryonic antigen (CEA), and alpha-fetoprotein (AFP) were all within normal parameters. 

**Figure 2:**
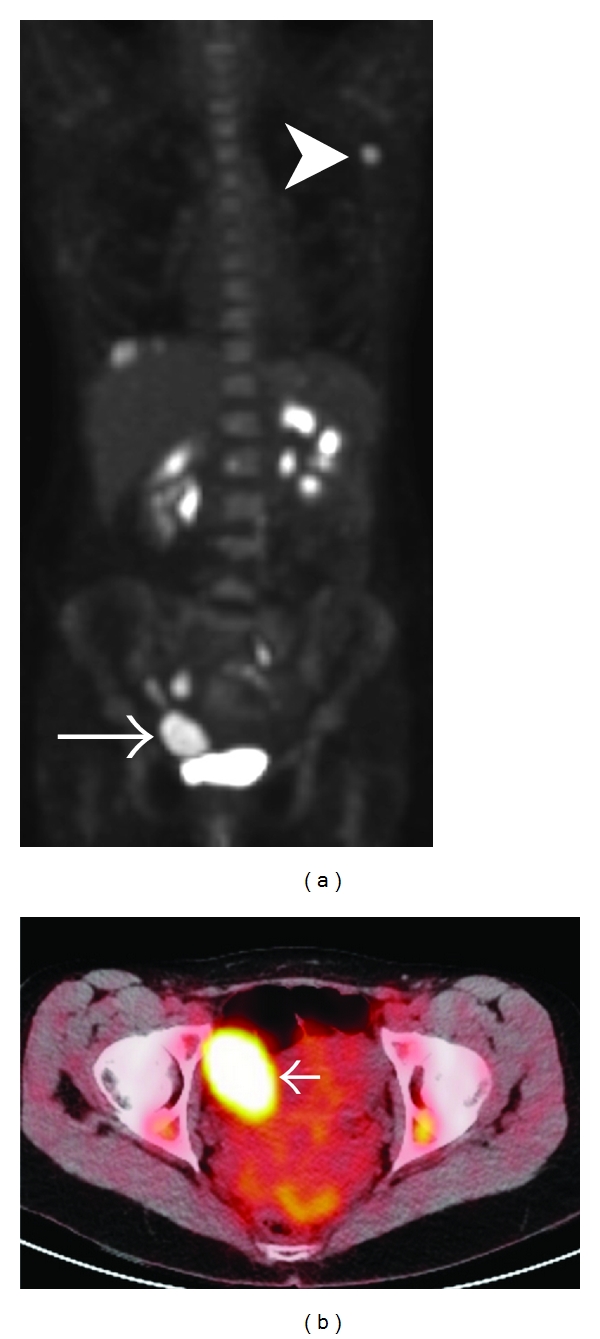
(a) Coronal maximum-intensity projection PET image shows abnormally increased FDG uptake within the left axillary lymph node (arrowhead) and within the right side of the pelvis (arrow). (b) Axial fused PET-CT image shows FDG-avid mass within the right adnexa of the pelvis, separate from the bladder (arrow).

A positron emission tomography-computed tomography (PET-CT) scan revealed a number of areas of high uptake, including the right pelvis, the left para-aortic region, and 2 small areas in the liver (Figure 2). A transabdominal US showed a normal anteverted uterus with 2 fibroids. A 3 cm left ovarian cyst was identified, but the right ovary was not well visualized. The patient proceeded to laparoscopy during which bilateral benign cystic ovaries were identified. The left Fallopian tube was normal, but the right fallopian tube was replaced by tumor and adherent to the bladder anteriorly. 

**Figure 3:**
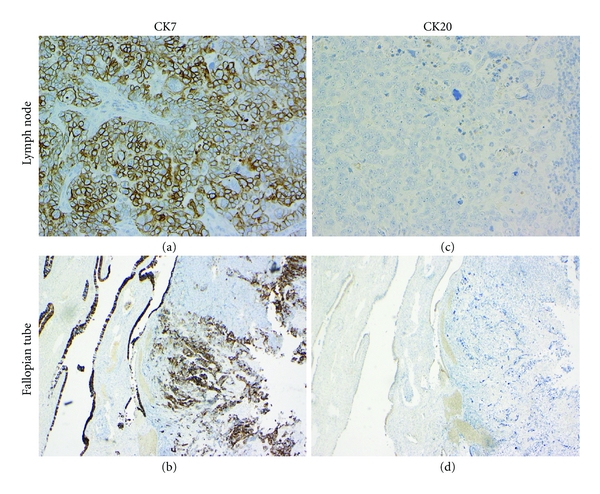
Crisp staining of tumour in the lymph node and the Fallopian tube with CK7 ((a) and (b)) and negative staining for CK20 for the same tumour at both sites ((c) and (d)).

**Figure 4:**
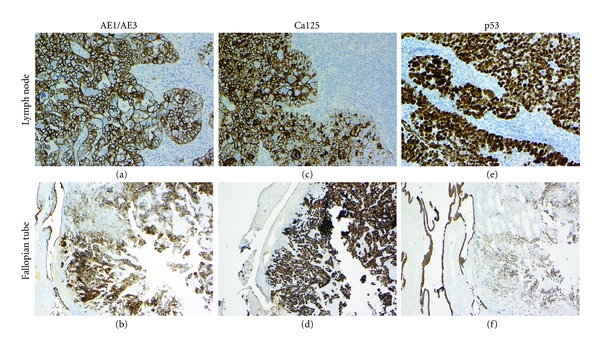
Membrane and cytoplasmic staining of tumour in the lymph node and the Fallopian tube with AE1/AE3 ((a) and (b)), Ca125 ((c) and (d)), and p53 ((e) and (f)).

A staging operation was undertaken, and a partial distal right salpingecomy was performed. Histology showed this to be extensively involved by a malignant high-grade Mullerian serous carcinoma with identical morphology to that found in the excised lymph node (Figure 1). Staining for CK7 was positive in both tissue samples, but negative for cytokeratin 20 (CK20) (Figure 3). In addition, similar to histology identified for the lymph node, The tumour in both the Fallopian tube and lymph node showed the same immunohistochemical profiles, CK7 positive, cytokeratin(CK)20 negative, cytokeratin AE1/AE3 positive, CA125 positive, and p53 positive (Figure 4). The tumour also showed nuclear positivity for Wilms tumour protein 1 (WT1) and focal positivity for placental alkaline phosphatise (PLAP) but was HCG negative. The histology was reviewed by 2 pathologists and sent for external expert pathology review which concluded that the tumour was a high-grade Mullerian serous carcinoma.

The patient underwent cytotoxic systemic chemotherapy, which consisted of 6 cycles of taxol and carboplatin. Following chemotherapy, she proceeded to have a total abdominal hysterectomy, bilateral salpingoopherectomy, and omentectomy. She had a good response to neoadjuvant therapy based on the rapid normalisation of tumour markers with no evidence of residual tumour visible at the time of surgery. She had an uneventful postoperative recovery. The patient, who is being followed at regular intervals, remains clinically well, with no evidence of disease recurrence or progression.

## 3. Discussion

To our knowledge, this is the first reported case of metastasis from an asymptomatic primary fallopian tube cancer (PFTC) to an axillary lymph node. Presentation with metastatic axillary node disease usually raises the suspicion of a primary breast tumor. However, in the absence of significant breast pathology, this can pose a diagnostic dilemma for the clinician. Enlargement of axillary lymph nodes is typically associated with malignancy in 35% of patients, the histology of which is usually lymphoma or adenocarcinoma [1]. Breast magnetic resonance imaging has proven useful in those patients with axillary lymphadenopathy, with no significant findings on US or mammogram, in identifying occult breast primaries [2]. Due to the lack of clarity regarding the location of the primary tumor following clinical, radiological, and histological examination of the enlarged axillary lymph node, a PET-CT using the radiotracer ^18^F-fluoro-2-deoxy-glucose (FDG) was performed. This mode of imaging has been shown to be more sensitive and specific than either computed tomography (CT) or MRI in determining the origin of unknown metastases [3]. As is evident in this case, it is of critical importance that the primary diagnosis is carefully established, in order to allow appropriate subsequent therapy.

 PFTC is extremely rare, with a reported incidence of 0.41 per 100,000 women [4]. PFTC malignancy typically spreads by intraperitoneal seeding, local invasion, or a combination of both. As a result, presentation with extra-abdominal lymphadenopathy, especially in the absence of widespread pelvic and intraabdominal disease, is a rare occurrence. Early PFTC is similar to ovarian cancer in that it is usually asymptomatic initially, and it is intraperitoneal spread that typically produces the symptoms and signs that are associated with the disease. The histological and clinical features of fallopian tube cancer are similar to those of ovarian cancer [5], and they appear to be identical under light microscopy [6]. It is therefore not surprising that some reports suggest that the actual incidence of PFTC may be underestimated, as advanced cases are often misdiagnosed as primary ovarian cancer [7]. In this case, based on the laparoscopic findings of normal ovaries with tumor invading the left fallopian tube, the diagnosis was considered more likely to be PFTC.

Histology of breast and ovarian or fallopian tube malignancies displays certain similarities and can pose a diagnostic challenge at the time of presentation of axillary lymph node metastasis. The use of immunocytochemical markers may prove useful in determining the origin of the tumor. Al-Hussaini et al. observed that 94.7% of ovarian serous carcinomas are positive for WT-1 [8]. In this current case, immunohistochemical testing with WT-1 was positive, indicating that a breast tumor was unlikely. Another useful marker to determine origin of the tumor is gross cystic disease fluid protein 15 (GCDFP-15), a protein isolated from breast cystic fluid, and may be used as a marker for breast cancer cells with apocrine differentiation. GCDFP-15 has a sensitivity of 74%, a specificity of 95%, and a positive predictive value of 74% for diagnosing breast cancer [9]. 56–93% of nonmucinous epithelial ovarian carcinomas express CA-125 on immunohistochemical testing [10, 11]. Whilst serum CA-125 levels are elevated in 80% of postmenopausal women with untreated nonmucinous epithelial ovarian carcinoma, levels are rarely elevated in breast cancer patients [12]. The elevated serum CA-125 and positive expression of CA-125 on immunohistochemical evaluation of fallopian tube in this instance strongly support the diagnosis of a gynecological malignancy.

PFTCs are typically managed with surgical staging, debulking, and adjuvant chemotherapy according to guidelines for treating ovarian carcinoma [6]. Surgery is the treatment of choice for PFTC, with an emphasis on aggressive tumor debulking with possible further surgery following or during chemotherapy. Residual disease >2 cm following surgery is considered a poor prognostic factor [13]. Staging of PFTC is similar to that of ovarian carcinoma, with a slight modification of the FIGO staging system. One-quarter of patients with PFTC have stage I disease, with the malignancy being confined to the fallopian tube, whilst 5–10% of patients present with stage IV disease with metastasis outside of the peritoneal cavity [14].

An important reason for distinguishing the origin of the primary tumor is to carefully tailor adjuvant therapy, as the potential different possible primary tumors require differing treatments. There is currently minimal literature regarding the optimal treatment for PFTC, but the current favored chemotherapeutic regimen for treating PFTC consists of a platinum-taxane regimen, as was used in this case [15]. In a study of patients treated with such a regimen, and who were adequately surgically debulked, the median progression free survival at 3 years was 67% [16]. Patients with stage IA or B disease with no tumor rupture pre- or intraoperatively may not require any adjuvant therapy. It is recommended, however, that patients with more advanced disease receive 3–6 cycles of carboplatin and paclitaxel [15].

A comprehensive review of 416 patients with PFTC revealed a 95% 5-year survival for stage I disease, 75% for stage II disease, 69% for stage III, and a 45% survival for stage IV disease [6]. A review of 35 cases of serous carcinoma of the ovary, fallopian tube of peritoneum presenting as lymphadenopathy revealed that there was no statistical difference in survival between those patients presenting with stage III or IV disease [17]. In addition, those patients with minimal intraperitoneal disease had a 5-year survival of 59%, indicating that this may represent a prognostically favorable subgroup. A number of negative prognostic factors are associated with PFTC including evidence of residual disease after surgery, depth of invasion of tumor into Fallopian tube, advanced stage, older age at presentation, poor histological differentiation of the tumor, Her2/neu overexpression, and elevated serum CA125 levels prior to commencement of treatment [5, 15, 18]. Fallopian tube cancer has been shown to have a strong genetic basis and has been linked to mutations in the BRCA1 and BRCA2 genes [19–21]. In patients with PFTC, a full history of malignancy within the family should be obtained and genetic counseling of the patient and relatives considered. Mutations of these genes and also alterations in p53 have also been shown to confer a worse outcome in PFTC [22, 23].

## 4. Conclusion

This case represents an unusual presentation of early primary fallopian tube cancer. It illustrates the importance of a high index of suspicion when dealing with axillary lympadenopathy with a clinically normal breast examination. It highlights the vital role of clinical, pathological, and radiological correlation in the diagnosis of such malignancies presenting in atypical circumstances.
